# Towards-Person Vocalization Effect on Screening for Autism Spectrum Disorders in the Context of Frustration

**DOI:** 10.3390/brainsci11121651

**Published:** 2021-12-16

**Authors:** Min Feng, Mengyao Zhai, Juncai Xu, Ning Ding, Nana Qiu, Huan Shao, Peiying Jin, Xiaoyan Ke

**Affiliations:** 1 Child Mental Health Research Center, The Affiliated Brain Hospital, Nanjing Medical University, Nanjing 210029, China; minfeng@njmu.edu.cn (M.F.); zhaimengyao@njmu.edu.cn (M.Z.); dingning@njmu.edu.cn (N.D.); qnn931210@hotmail.com (N.Q.); linyan92@njmu.edu.cn (H.S.); hanlu@njmu.edu.cn (P.J.); 2 Case School of Engineering, Case Western Reserve University, Cleveland, OH 44106, USA; juncai.xu@gmail.com

**Keywords:** autism spectrum disorder, infants, still-face paradigm, vocalization

## Abstract

The purpose of this study is to investigate the vocalization characteristics of infants with autism spectrum disorder (ASD) in the context of frustration. The duration and frequency of vocalization in 48 infants with ASD and 65 infants with typical development (TD) were followed up to 24 months later for subsequent diagnosis. The typical vocalizations of infants with ASD were retrospectively analyzed, such as speech-like vocalizations, nonspeech vocalizations, vocalizations towards the person and non-social vocalizations. The results showed that, compared with the TD group, vocalizations of infants with ASD during the still-face period had lower typical vocalizations and characteristics associated with social intention, and that these characteristics were closely related to the clinical symptoms of ASD, among which vocalizations towards the person accompanied by social intention had discriminative efficacy.

## 1. Introduction

Autism spectrum disorder (ASD) is a neurodevelopmental disorder characterized by deficits, limitations and repetitive interest behavior patterns in social interaction and communication [1]. The prevalence of ASD has been increasing in recent years and is about 1.5% in the general population [2]. The prevalence rate of ASD is as high as 10–20% in high-risk groups, such as siblings with ASD [3,4]. At present, the pathological mechanism of ASD is unknown and there is no specific drug treatment. Early detection and diagnosis can promote early intervention and thus effectively improve the prognosis of children with ASD [5,6,7]. Although the diagnosis of ASD was stable at 2 years of age [8,9], a recent study found that the diagnostic stability of ASD was 79% at 14 months and 83% at 16 months [10]; however, in reality the average age of diagnosis is after 3 years [11]. Therefore, research on early behavioral indicators of ASD is of great significance to promote the early detection and diagnosis of ASD.

The early abnormal behavior of ASD can be observed as early as 12 months of age but the heterogeneity is significant and no single behavioral indicator has been found to predict a stable diagnosis outcome of ASD. The early abnormal behavior of ASD mainly showed difficulties in social communication and stereotyped behavior [12,13]. Studies have found that nearly 60% of children aged 4–6 years with ASD have moderate to severe language problems [14]. An abnormal language development trajectory was usually the earliest abnormal behavior reported by parents of children with ASD, with 56% of parents showing concern about their children’s insufficient language communication ability as early as 10–16 months of age [15], and language ability in childhood is one of the strongest predictors of a long-term prognosis of ASD [16]. Language impairment is an essential behavioral indicator of clinical symptoms and prognosis of ASD. It is also a significant cause of parents’ early concern and consultation. Therefore, the study of early language and phonological behavior of ASD is of great value to the early behavioral indicators of ASD.

Children with typical development (TD) usually have a typical language development trajectory, with the appearance of speech-like vocalizations containing canonical syllables at 6–10 months of age, such as “babbling”, and more diverse “babbling” at 10–12 months of age, gradually evolving into the first word [17,18]. Children with ASD typically have abnormal vocalizations: a lower proportion of speech-like vocalizations and higher proportion of nonspeech-like vocalizations [19,20,21,22,23]. The still-face paradigm (SFP) was first proposed by Tronick et al. in 1978 to evaluate how infants and young children respond to social interactions and social accidents and to prove that infants and young children are the primary contributors to social interaction [24]. Classic SFP contains three episodes: the baseline episode (normal interaction between mother and child); the still-face (SF) episode (the mother maintains a neutral facial expression and does not respond to the child’s behavior); the reunion episode (the mother recovers her interaction with the child). At present, the SFP has been applied in the study of emotion regulation ability, social interaction behavior and social expectation of children aged 4–36 months [25,26,27]. In the SF episode, when mothers stop the interaction and show no expression, children usually show a typical still-face effect, which is mainly manifested by a typical social stress response and increased negative emotional behavior [28,29,30]. Studies on early vocalizations using the SFP have found that normal children’s SF episode is an effective frustration situation. It shows significantly different vocalizations to try to re-awaken the mother’s interaction, and the change of vocalizations was observed in the first 3 months [31,32]. However, no studies on the use of the SFP to explore the vocalizations of children with ASD have been found.

This study explores the early vocalization characteristics of High Risk-ASD (HR-ASD) children through the SFP. It explores the relationship between these vocalization characteristics and age, development level and clinical symptoms of ASD to provide theoretical support for seeking behavioral indicators of an early diagnosis of ASD.

Based on social communication ability as the core symptom impairment of ASD, we assumed that the Still Face episode (SF) could be used as a frustration situation to highlight the abnormal characteristics of ASD vocalization behavior, especially that accompanying social intention vocalization behavior. Therefore, this study compared and analyzed differences in typical vocalizations and vocalizations towards an object/person in the HR-ASD group with ASD diagnosis (symptoms consistent with ASD diagnosis but less than 24 months of age or ASD sibling) and the TD group in SF episode to study the indicators of early vocalizations with more distinguishing efficiency.

## 2. Research Object and Method

### 2.1. The Research Object

From January 2018 to January 2020, 66 siblings with undiagnosed ASD symptoms or ASD were enrolled into the HR-ASD group (mean age 18 months; male 71%) and 65 children with normal development were recruited into the TD group (mean age 12 months; male 55%) during the same period. Four researchers followed infants in the HR-ASD group up to 24 months of age. The diagnostic evaluation was carried out using the Autism Diagnostic Observation Schedule (ADOS) and the Childhood Autism Rating Scale (CARS), and clinical diagnosis was made by two senior child psychiatrists according to the diagnostic criteria of ASD in the Diagnostic and Statistical Manual of Mental Disorders, Fifth Edition (DSM-5). During follow-up, 16 cases did not meet the diagnosis of ASD and 2 cases were lost to follow-up. Finally, 48 infants with ASD remained in the ASD group.

At the time of enrollment, HR-ASD met the following requirements: the Modified Checklist for Autism in Toddlers (M-CHAT) was positive; diagnosis according to the diagnostic criteria of ASD in the DSM-5 by two senior child psychiatrists [1]; the age ranges from 6 to 23 months; and the primary caregiver is the mother; not participate in any treatment. Exclusion criteria were: genetic or metabolic diseases with clear etiology; all kinds of neurodevelopmental disorders except ASD; and history of craniocerebral trauma, neurological diseases and severe physical diseases; hearing impairment. The TD group inclusion criteria were age 7–23 months and the primary caregiver is the mother. Exclusion criteria were all kinds of neurodevelopmental disorders and mental disorders and a history of craniocerebral trauma, neurological disease and physical disease.

The Medical Ethics Committee of the Brain Hospital affiliated to Nanjing Medical University (2017-KY098-01) approved this study and informed consent was obtained from the parents of the children.

### 2.2. Research Tools

The modified checklist for autism in toddlers [33] (M-CHAT) and the Communication and Symbolic Behavior Scales Development Profile Infant-Toddler Checklist (CSBS-DP-ITC) were used for early HR-ASD symptom screening in this study. The Gesell Developmental Checklist (GDC) [34] was used to assess development level and the Childhood Autism Rating Scale (CARS) [35] and Autism Diagnostic Observation Schedule (ADOS) [36] were used to evaluate clinical symptoms of ASD, as shown in Table 1.

### 2.3. Vocalization Acquisition and Coding

**Figure 1:**
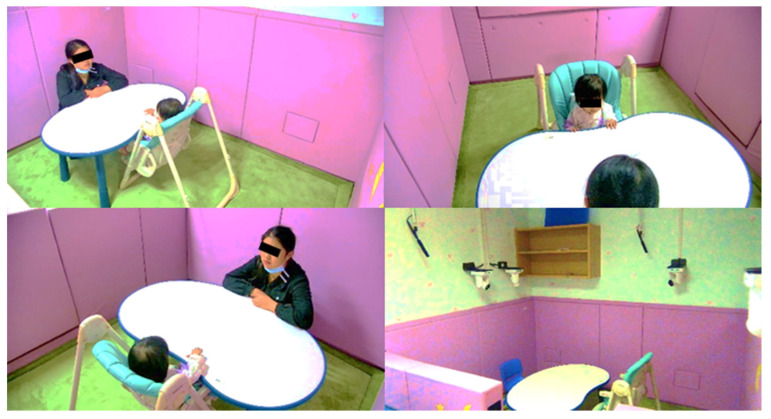
The still-face paradigm (SFP) behavioral video collection settings.

Vocalizations were captured using the SFP behavioral video in a standardized behavior observation room. The researchers unified instructions and environment settings. Infants were arranged to sit on fixed dining chairs and mothers were arranged to sit opposite and interact with infants for 2 min without toys or physical contact (interaction period). Two minutes is the baseline period for vocal collection. Then they stopped the interaction and mothers looked straight at the infants’ heads and maintained neutral facial expressions for 1 min (static period) (Figure 1).

Based on the behavioral coding system of children developed by Werner and Dawson and the coding definition of vocalizations in previous studies [37], vocalization behavior indicators in this study included two types:

(1) Typical vocalizations: speech-like vocalizations that contain standardized syllables such as babbling; and nonspeech-like vocalizations of words and syllables that cannot be recognized, mainly including three types–sad vocalizations (e.g., crying), a happy sound (e.g., laughter) and atypical vocalizations (e.g., screams).
(2) Vocalizations towards object/person: vocalizations towards a person have social intention, such as gaze at others’ faces, or with pointing, giving, sharing and other meanings; in contrast, with vocalizations towards an object there is no social intention.

Two trained professionals coded the duration and frequency of the above two types of vocalizations using the Observer XT 12.0 (an analysis system for behavior observation recording) in the SF episode. The two types of 140 vocalizations were randomly assigned to two trained coders to control evaluator bias. The two coders did not participate in the SFP experiment. Both of them were blind to the grouping of the coded children. Vegetative sounds such as yawning, burping and coughing were excluded from the analysis [38]. In this study, 20% of the videos were randomly selected as a coder consistency test. Through intraclass correlation coefficients (ICC), it was found that the coding consistency coefficients of the two graduate students were all in the good to excellent range: (1) typical nonspeech-like vocalizations: duration = 0.897 s, frequency = 0.680; speech-like vocalizations: duration = 0.976 s, frequency = 0.969; (2) vocalizations towards person: duration = 0.830 s, frequency = 0.701; vocalizations towards object: duration = 0.816 s, frequency = 0.914.

### 2.4. Statistical Treatment

SPSS 23.0 was used for the statistical data analysis. In this analysis, gender is expressed by the chi-square test of the case (%). The Shapiro–Wilk test was used as a normality test. Measurement of normally distributed data were expressed as the mean ± standard deviation ($\bar{\chi }$ ± *s*) and an independent sample *t*-test was used for intergroup comparison. The measurement data of non-normal distribution were expressed as the median (interquartile spacing) [*M*(*P*_25_, *P*_75_)] and comparison between groups was performed by the Mann–Whitney U test. Pearson’s correlation analysis was used to explore the relationship between vocalization behavior and age, development level and clinical symptoms of ASD (*p* < 0.05 was considered to be statistically significant). A binary logistic regression model was used for regression analysis and a multilayer perceptron (MLP) was used to build an early screening model for ASD.

### 2.5. Experimental Process

**Figure 2:**
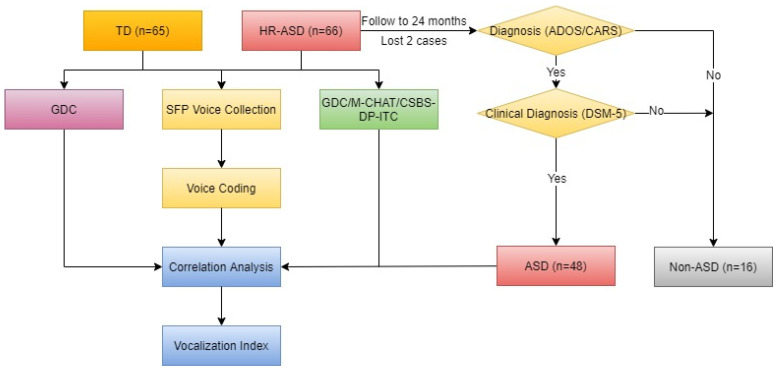
Flow chart of ASD vocalization experiment.

First, both groups completed development-level assessment and SFP behavioral test voice collection and the HR-ASD group additionally completed ASD symptom assessment. Four professionals followed the HR-ASD group up to 24 months to complete the diagnostic assessment of ASD. If assessed as ASD, the diagnosis was made by two senior child psychiatrists according to the DSM-5. From the development, clinical symptom and voice data of the groups, the characteristics of ASD vocalization behavior were analyzed, the relationship between ASD vocalization behavior and clinical symptoms was studied and the indicators of vocalization behavior with distinguishing efficiency were determined (Figure 2).

## 3. Results

### 3.1. Comparison of General Data between the ASD and TD Groups

There were statistically significant differences in age and developmental quotient (DQ) (adaptive, fine-motor, language and personal-social) between the ASD and TD groups (*p* < 0.05) but there were no statistically significant differences in gender or grand motor DQ (*p* > 0.05), as shown in Table 2.

### 3.2. Comparison of Static Vocalization Behavior in the ASD and TD Groups

**Figure 3:**
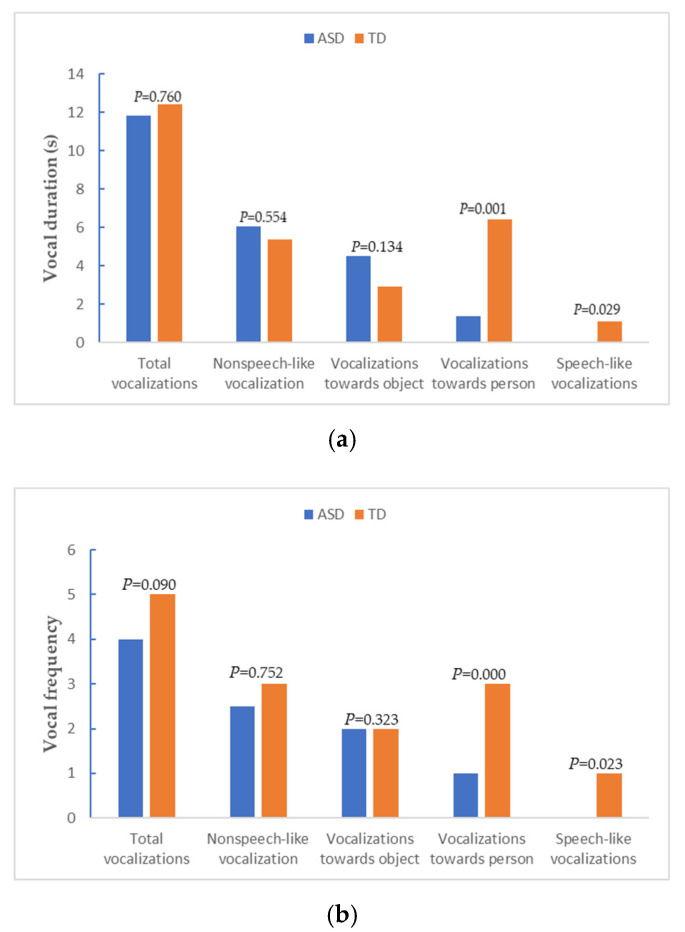
Comparison of SFP vocalizations between the ASD and TD groups: (**a**) vocal duration; (**b**) vocal frequency.

In the SF episode, on comparing the ASD and TD group SFP vocalization results (Figure 3a,b), the duration and frequency of speech-like vocalizations and vocalizations towards the person in the TD group were higher than those in the ASD group; the difference was statistically significant (*Z* = −2.183, −3.179, −2.275, −3.707; *p* < 0.05). However, total vocalizations, nonspeech-like vocalization and duration and frequency of vocalizations towards an object have no statistical significance in the two groups (*p* > 0.05).

### 3.3. Correlation Analysis of SFP Vocalizations with Age, DQ and Clinical Symptoms in the ASD Group

According to the correlation analysis results of SFP vocalizations with age, development level and clinical symptoms (Table 3), the duration and frequency of vocalizations towards the person in the ASD group were positively correlated with adaptive DQ (*p* < 0.05) and the frequency of vocalizations towards person was positively correlated with fine motor DQ (*p* < 0.05). There was a positive correlation between the duration of speech-like vocalizations and age (*p* < 0.05) but no significant correlation between other indices and age or DQ (*p* > 0.05).

The duration and frequency of vocalizations towards the person were negatively correlated with the social communication and total score of the CARS and ADOS (both *p* < 0.05). The frequency of vocalizations towards the person was also negatively correlated with the limited and rigid behavior of the ADOS (*p* < 0.05). The duration of speech-like vocalizations was positively correlated with symbolic behavior in the CSBS-DP-ITC (*p* < 0.05). Its duration and frequency were also positively correlated with social communication, language factor and total score of the CSBS-DP-ITC (*p* < 0.05). However, the total vocalization frequency of ASD was negatively correlated with the CARS (*p* < 0.05). There was no significant correlation between other indicators and clinical symptoms (*p* > 0.05).

### 3.4. Regression Analysis of ASD Screening in SFP Static Vocalizations

The binary logistic regression equation was constructed by including speech-like vocalizations and the duration and frequency of vocalizations towards the person. Binary logistic regression analysis of the ASD diagnosis in the SF episode showed that the frequency of vocalizations towards the person had discriminating efficiency (OR = 1.609, 95% CI = 1.143–2.266; *p* = 0.006) (Table 4).

### 3.5. Use of an MLP to Build an Early Screening Model for ASD

The length of vocalizations towards the person, the age and the length and frequency of speech-like vocalizations in the SF episode were taken as sample behavioral characteristics. The ASD and TD groups were taken as samples, with the ASD group labeled as 0 and the TD group as 2; 70% of the data were randomly selected as the training set and 30% as the test set. The results show that classification accuracy was 93% in the ASD group and 75% in the TD group, with an average accuracy of 84% (Table 5).

**Figure 4:**
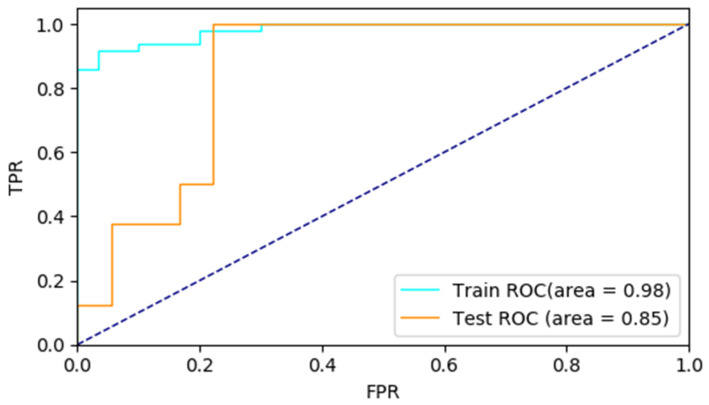
ROC of the multilayer perceptron classifier.

The receiver operating characteristic curve (ROC) is a graphical plot that illustrates the diagnostic ability of a binary classifier system as its discrimination threshold is varied. The ROC of the proposed MLP is shown in Figure 4. The true positive rate (TPR) changes with false positive rate (FPR) in the Figure 4. The area under the curve (AUC) is equal to the probability that a classifier will rank a randomly chosen positive instance higher than a randomly chosen negative one. It can be found the proposed MLP shows a good recognition performance as AUC is greater than 0.75.

## 4. Discussion

In the study, a comparative analysis of ASD and TD groups showed that there were indeed abnormal early vocalizations in ASD, and abnormal vocalizations had significant potential in the early screening of ASD. The length and frequency of speech-like vocalizations towards the person in the SF episode for the ASD group are less than for the TD group. The TD group showed more paralanguage and vocalizations towards the person with social intention, such as pointing, sharing and giving, indicating that the TD group would use more active social behaviors to attract and interact with their mothers in a frustrating situation. In contrast, the ASD group showed lower social motivation in a frustrating situation, which suggests that frustrating situations are more likely to highlight impaired co-attention-based active social interactions in infants with ASD. This study is consistent with the findings of Yirmiya et al., where infants with ASD already show less requesting behavior and social participation behavior before 14 months of age [39]. The results of this study support the exploration of a combination of language maturity and social intention as an early behavioral indicator of ASD [40]. There is a social feedback loop between children’s language development and adults’ vocalization. Adults tend to respond to children’s typical vocalizations and the adults’ responses influence children’s vocalizations. Children with ASD have more nonspeech-like vocalizations and vocalizations towards the object, and these atypical vocalizations are easily ignored by adults, which leads to fewer responses from adults.

Meanwhile, impairment of the social core of ASD prevents them from learning from the fewer responses from adults. The incomplete social feedback loop between children with ASD and adults further deprives them of early language and social learning opportunities, thereby exacerbating the core damage of ASD [41]. Therefore, studying the behavioral characteristics of early ASD vocalization is of great significance for exploring the damage of early ASD core symptoms, providing valuable behavioral indicators for early detection and exploring the critical goals of early clinical intervention. In recent years, it has been a trend to combine the maturity of language expression (typical vocalizations) and vocalizations of social intention to find more reliable early behavioral indicators of ASD vocalizations [42]. In this study, the phonological behaviors of children with ASD in the context of frustration, especially those with social intention, are more prominent than those in the context of normal interaction.

Although the average duration of vocalizations towards the object in the ASD group was longer than in the TD group, the difference between the two groups was not statistically significant. This is consistent with previous literature. Ozonoff et al. found that the vocalizations of infants with ASD at 12–36 months of age were accompanied by a lower gaze but there was no abnormality in their vocalizations toward objects at 6–36 months of age [43]. In normal social situations, such as free games or frustrating situations, infants with ASD have abnormal vocalizations towards the person but there is no significant difference in vocalizations towards objects compared with TD infants. These results suggest that vocalizations towards objects may not play an early role in discrimination.

It is still controversial whether the typical vocalizations of children with ASD are abnormal [40,42]. Chenausky et al. believed that children with ASD had significantly less speech-like vocalizations than TD infants in the early stage [22] but other researchers held the opposite view that there was no difference [44]. The results of this study support the former view. By comparing the vocalizations of infants in the ASD and TD groups in a frustrating situation, it was found that the duration and frequency of vocalizations in the ASD group were less than in the TD group; however, there was no significant difference in nonspeech-like vocalizations between the two groups. These results suggest that children with ASD have typical abnormal vocalizations in the context of frustration, which is mainly manifested by less speech-like vocalizations, and that nonspeech-like vocalizations may not play a discriminative role.

Correlation analysis showed a positive correlation between the duration and frequency of speech-like vocalizations with age, social communication, language factor and symbolic behavior, but no correlation with the scores of CARS and ADOS. It indicates that speech-like vocalizations reflect the development process of typicality. The duration and frequency of vocalization towards person were correlated with the scores of CARS and ADOS, which indicated that the vocalization towards person reflected social indicators. The duration and frequency of vocalization towards person were correlated with individual indicators in the developmental scale, and the results were not representative. There was no correlation between other vocalization behavior, age and developmental level.

ASD vocalizations were correlated with clinical symptoms, language and social development ability to varying degrees and that the duration and frequency of vocalizations towards the person were negatively correlated with the social core symptoms and limited stereotyped behavior of ASD. There is a positive correlation between the duration and frequency of speech-like vocalizations and the development of children’s social communication and language competence but no correlation with age or other developmental dimensions. The lower the total vocal frequency of children with ASD, the higher the symptom score, and vice versa. There was no correlation between nonspeech-like vocalizations and clinical symptoms, which is consistent with Chericoni’s results [45]. The results of this study support vocalizations (especially their length and frequency) towards the person as potentially valuable behavioral indicators and speech-like vocalizations as reflecting whether the language and cognitive development of infants with ASD is delayed. Regression analysis further found that the frequency of vocalizations towards the person had discriminative efficacy and that vocalizations towards the person with social intention could predict the diagnosis of ASD better than typical vocalizations in frustrating situations. The results of this study suggest that vocalization with social intention is more valuable for the early identification and screening of core symptoms in infants with ASD than typical vocalizations in the context of frustration. An MLP was used to construct an early screening model for ASD based on the behavioral characteristics of targeted vocalization, age and duration and frequency of speech-like vocalizations in the context of frustration, and the accuracy of predicting the outcome of ASD was up to 84%.

## 5. Limitations and Future Directions

There are several limitations to the current study. Firstly, the age of HR-ASD group was higher than that of TD group, we found that the age with most indicators of vocalization behavior does not exist in correlation. Secondly, environmental and intervention factors that may affect infant vocalization were not included in this study, and 16 HR-ASD children who no longer meet the diagnosis of ASD during follow-up may have false-negative cases. Moreover, this study is a preliminary study of ASD vocalization. Vocalizations towards person in this study have a certain effectiveness in predicting ASD. However, it is not the only diagnosis predictor. Future more prolonged studies include the suspected but not confirmed ASD, analyzing the family environment and intervention factors to evaluate the utility of the ASD predictors better. 

## 6. Conclusions

This study showed that children with ASD had abnormal vocalizations at an early stage, manifesting in both typical vocalizations and vocalizations towards people with social intention. The former may be related to the language development process of children whereas the latter are often closely related to the severity of the clinical symptoms of ASD and have an important differential significance for predicting the outcome of ASD. Parents’ concerns and reports on the vocalizations of infants with ASD have a certain clinical basis that should be taken into account in early screening.

According to this study, vocalizations towards people and typical vocalizations are of great significance for ASD children. Parents should pay attention to providing a rich language environment, such as imitation and timely response to the children’s vocalizations, guiding the joint attention in the parent-child interaction, and reinforcing children’s vocalizations towards people to promote the ASD children’s language development and improve social interaction.

## Figures and Tables

**Table 1:** Assessment tools for ASD vocalization experiment.

Evaluation Indicators	M-CHAT	GDC	CSBS-DP-ITC	ADOS	CARS
Early symptom screening	√		√		
Assessment of development level		√			
Evaluation of clinical symptoms				√	√

ASD, autism spectrum disorder; M-CHAT, The modified checklist for autism in toddlers; GDC, Gesell Developmental Checklist; ADOS, Autism Diagnostic Observation Scale; CSBS-DP-ITC, Communication and Symbolic Behavior Scales Development Profile Infant-Toddler Checklist; CARS, Childhood Autism Rating Scale.

**Table 2:** Comparison of general data between the ASD and TD groups [$\bar{\chi }$ ± *s* or *M*(*P*_25_, *P*_75_)].

Project	ASD Group (*n* = 48)	TD Group (*n* = 65)	*χ*^2^(*t*) [*Z*]	*p* Values
Gender (Male/Female)	34/14	36/29	2.795	0.095
Age (months)	18.00 (11.25, 21.00)	12.00 (12.00, 18.00)	−3.881	0.000
DQ				
Adaptive	82.29 ± 2.46	93.49 ± 1.15	−4.475	0.000
Grand motor	90.48 ± 2.36	95.00 (88.00, 98.00)	−0.893	0.372
Fine motor	88.30 ± 2.44	96.00 (92.60, 100.00)	−2.620	0.009
Language	69.50 (50.00, 87.00)	92.00 (84.00, 100.00)	−5.850	0.000
Personal-social	83.00 (71.50, 91.00)	92.00 (86.00, 102.00)	−4.324	0.000
ADOS				
Social interaction	13.46 ± 4.13	-	-	-
Stereotypes and limited interests	2.00 (1.00, 2.75)	-	-	-
CSBS-DP-ITC				
Social interaction factor	12.44 ± 4.31	-	-	-
Language factor	5.40 ± 3.03	-	-	-
Symbolic act	7.85 ± 3.89	-	-	-
CARS	32.04 ± 5.36	-	-	-

ASD, autism spectrum disorder; TD, typical development; DQ, developmental quotient of the Gesell Developmental Checklist; ADOS, Autism Diagnostic Observation Scale; CSBS-DP-ITC, Communication and Symbolic Behavior Scales Development Profile Infant-Toddler Checklist; CARS, Childhood Autism Rating Scale.

**Table 3:** Correlation analysis of SFP vocalizations in the ASD group with age, development level and clinical symptoms (*r* value).

Vocalization Behavior Index	Age (Months)	DQ					CSBS-DP-ITC				CARS		ADOS	
		Adaptive	Grand Motor	Fine Motor	Language	Personal-Social	Social Communication	Language Factor	Symbolic Behavior	Total Score		Social Interaction	Limited and Rigid Behavior	Total Score
Vocal duration (s)														
Total vocalizations	0.072	0.181	0.053	0.020	0.128	−0.061	0.099	0.110	0.005	0.085	−0.218	−0.227	−0.192	−0.250
Nonspeech-like vocalization	−0.047	0.135	0.045	0.026	0.109	−0.041	−0.012	−0.028	−0.118	−0.055	−0.189	−0.219	−0.176	−0.239
Speech-like vocalizations	0.318 *	0.140	0.025	−0.015	0.064	−0.062	0.305 *	0.377 **	0.322 *	0.379 **	−0.102	−0.048	−0.061	−0.059
Vocalizations towards person	−0.053	0.346 *	0.254	0.251	0.139	0.188	0.074	−0.053	−0.120	−0.016	−0.394 **	−0.300 *	−0.180	−0.310 *
Vocalizations towards object	0.127	−0.003	−0.106	−0.146	0.061	−0.208	0.087	0.161	0.085	0.119	−0.018	−0.090	−0.118	−0.110
Vocal frequency (frequency)														
Total vocalizations	0.127	0.179	0.045	0.075	0.090	−0.067	0.147	0.093	0.098	0.130	−0.320 *	−0.213	−0.242	−0.251
Nonspeech-like vocalization	−0.078	0.122	−0.101	0.012	0.014	−0.102	−0.068	−0.147	−0.068	−0.107	−0.276	−0.213	−0.268	−0.259
Speech-like vocalizations	0.268	0.116	0.163	0.058	0.131	0.003	0.294 *	0.324 *	0.229	0.322 *	−0.161	−0.082	−0.043	−0.083
Vocalizations towards person	−0.088	0.382 **	0.215	0.357 *	0.240	0.271	0.190	0.024	−0.009	0.091	−0.535 **	−0.347 *	−0.304 *	−0.385 **
Vocalizations towards object	0.295 *	−0.064	−0.084	−0.157	-0.109	−0.279	0.040	0.102	0.158	0.109	0.019	0.022	−0.106	−0.010

SFP, still-face paradigm; ASD, autism spectrum disorder; DQ, developmental quotient of the Gesell Developmental Checklist; ADOS, Autism Diagnostic Observation Scale; CSBS-DP-ITC, Communication and Symbolic Behavior Development Scale Infant -Toddler Checklist; CARS, Childhood Autism Rating Scale; * *p <* 0.05; ** *p <* 0.01.

**Table 4:** Binary logistic regression analysis of ASD diagnosis in the still-face episode.

Vocalization Index	*B*	SE	*p*	OR	95% CI	
					Upper Limit	Lower Limit
Vocal duration (s)						
Speech-like vocalizations	0.001	0.067	0.993	1.000	0.878	1.140
Vocalizations towards person	0.055	0.043	0.195	0.946	0.871	1.029
Vocal frequency (secondary)						
Speech-like vocalizations	0.037	0.170	0.829	0.964	0.691	1.344
Vocalizations towards person	0.476	0.175	0.006	1.609	1.143	2.266

ASD, autism spectrum disorder; *B*, partial regression coefficient; SE, standard error; OR, odds ratio; CI, confidence interval.

**Table 5:** Data table of model prediction results.

Data Category		Precision	Recall	F1-Score	Support
Train data	ASD	0.97	0.93	0.95	30
	TD	0.96	0.98	0.97	49
	Average	0.96	0.96	0.96	39.5
Test data	ASD	0.93	0.72	0.81	18
	TD	0.75	0.94	0.83	16
	Average	0.84	0.83	0.82	17
